# Changes in intraocular pressure after clear corneal phacoemulsification in normal patients

**DOI:** 10.4103/0974-620X.57309

**Published:** 2009

**Authors:** Salima Bhallil, Idriss Benatiya Andalloussi, Fouad Chraibi, Khadija Daoudi, Hicham Tahri

**Affiliations:** Department of Ophthalmology, University Hospital, Hassan II, Fez, Morocco

**Keywords:** Clear corneal phacoemulsification, intraocular pressure, non-glaucomatous patients

## Abstract

**Purpose:**

To evaluate changes in intraocular pressure (IOP) after clear corneal phacoemulsification (CCP) in normal patients.

**Materials and Methods:**

A prospective study including 273 normal patients selected for cataract extraction by CCP. Intraocular pressure was recorded on the 15^th^ day, l^st^, 2^nd^, 3^rd^ month and 6 months after surgery.

**Statistical Analysis:**

For statistical analysis, Epi Info was used to determine the statistical significance of changes in IOP.

**Results:**

The mean age of 96 women and 177 men was 71 ± 12 years. The mean IOP before surgery was 14.18 ± 3.4 mmHg. Our patients showed a mean decrease in IOP of 2.25 mmHg (16%) compared to preoperative values. Change in IOP was not related to lens thickness (*P* = 0.12), but significantly correlated with change in anterior chamber depth (ACD) (*P* = 0.002). The postoperative IOP was inversely related to preoperative ACD (*P* = 0.012). Age, sex and axial length were not significantly related to IOP reduction (*P* = 0.2–0.5)

**Conclusion:**

CCP was associated with a statistically significant reduction in IOP. The exact mechanism by which cataract surgery results in IOP reduction is unclear. CCP can be performed with the intent of achieving better IOP control.

## Introduction

Cataract extraction surgery, independent of the technique used, induces variations in intraocular pressure (IOP). Although an elevation of the IOP in the early postoperative stage may be noted, many studies have reported a reduction in IOP.[1‐3] Some studies have reported that cataract extraction widens the angle, deepens the anterior chamber and leads to a significant reduction of IOP, particularly in eyes with narrow angles.[4,5] The purpose of this study was to examine changes in IOP after uneventful clear corneal phacoemulsification (CCP) in normal patients.

## Materials and Methods

This was a prospective study including 273 normal patients selected for cataract extraction by phacoemulsification using a 3.2 mm clear corneal incision between June 2003 and January 2006. The study was approved by the Hassan II University Ethics Committee.

A prospective analysis was performed using clinical charts focusing on patient age and sex, size of the capsulorhexis, and pre- and postoperative IOP. The axial length, lens thickness and anterior chamber depth (ACD) were measured during preoperative assessment three weeks before surgery using ultrasound A scan by contact technique. Central corneal thickness was not assessed. IOP was measured by Goldmann applanation tonometer by the same examiner preoperatively, on the 15th day, and subsequently one, two, three and six months after surgery. The mean change in IOP after surgery was calculated. The mean follow-up period was six months.

Patients with history of ocular surgery, trauma, preoperative IOP greater than 21 mmHg, on ocular medication and who developed postoperative complication were excluded from the study.

### Statistical analysis

For statistical analysis, Epi Info was used to determine the statistical significance of changes in IOP. The statistical significance between the groups was estimated using Chi test[2]. A value of <0.05 was considered significant. Correlation coefficients were also estimated using Pearson′s test.

### Surgical technique

All surgeries were performed by the same surgeon.

Most of the patients underwent surgery under peribulbar anesthesia using Lidocaine.

Surgery involved a 3.2 mm superior clear corneal tunnel incision, injection of viscoelastic material into the anterior chamber, capsulorhexis of 5 mm, hydrodissection, in the bag phacoemulsification using phaco-chop technique, cortex aspiration, additional injection of viscoelastic material and insertion of foldable hydrophobic intraocular lens (IOL) in the capsular bag. The viscoelastic material was then removed. The corneal incision was closed by stromal hydration.

Postoperatively, all patients were treated with topical combination of dexamethasone and Neomycin eyedrops during four weeks, and topical nonsteroidal antiinflammatory eyedrops four times daily for eight weeks.

## Results

**Figure 1:**
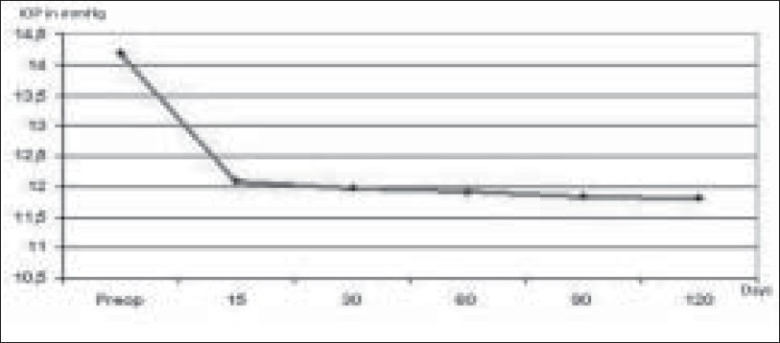
Mean IOP after phacoemulsification. The mean IOP was 12.07 ± 2.6 mmHg 15 days, 11.98 ± 3.1 mmHg 1 month, 11.92 ± 2.4 mmHg two months, 11.84 ± 1.4 mmHg 3 months and 11.82 ± 1.3 mmHg six months after phacoemusification

Two hundred and seventy three eyes of 273 patients were recruited for the study. The mean age of the 96 women and 177 men was 71 ± 12 years. The mean IOP before surgery was 14.18 ± 3.4 mmHg. Mean preoperative ACD was 2.96 mm and postoperative ACD was 4.09 mm. The mean lens thickness was 4.24 mm and axial length was 23 mm. A postoperative reduction of IOP was found as shown in Figure 1. After 15 days, the mean IOP was 12.07 ± 2.6 mmHg (*P* = 0.012), after 1 month 11.98 ± 3.1 mmHg (*P* = 0.015), after two months 11.92 ± 2.4 mmHg (*P* = 0.005), after 3 months 11.84 ± 1.4 mmHg and after six months 11.82 ± 1.3 mmHg (*P* = 0.005).

**Figure 2:**
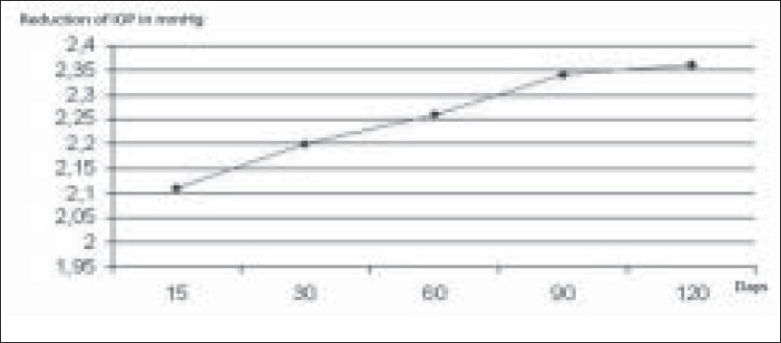
Mean reduction IOP after phacoemulsification. Compared to preoperative values, our group had a mean decrease in IOP of 2.25 mmHg

**Figure 3:**
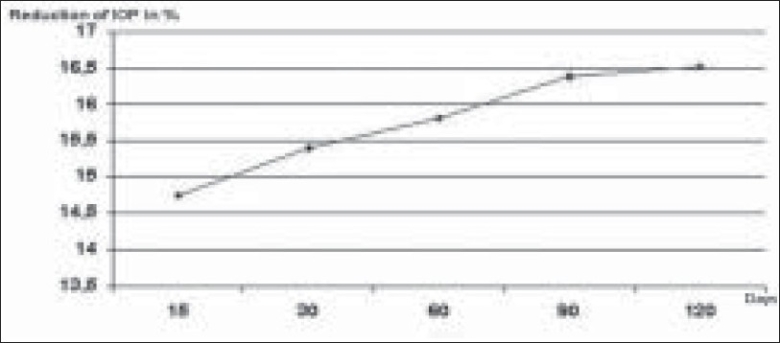
Reduction of IOP in % after phacoemulsification. Compared to preoperative values our group had a mean decrease in IOP of 15.77%

The postoperative reduction in IOP in mmHg and in percentage is shown in Figures 2 and 3.

The reduction of IOP measured after 15 days was 2.1 mmHg, after 1 month 2.26 mmHg, after three months 2.34 mmHg and after six months 2.36 mmHg. Our group had a mean decrease in IOP of 2.25 mmHg (15.77%) compared to preoperative values (*P* = 0.011). Lens thickness was not significantly related to change in IOP (*P*: 0.12); however, it was significantly related to change in ACD (*P*: 0.002). The postoperative IOP was inversely related to preoperative ACD (*P*: 0.012). Age, sex, axial length were not significantly related to IOP reduction range of *P*: 0.2–0.5.

## Discussion

**Figure 4:**
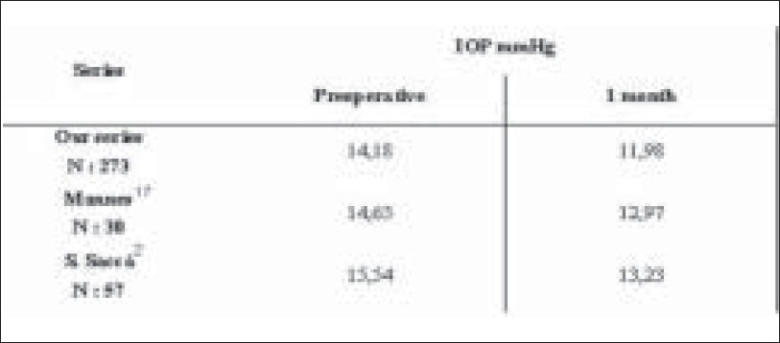
Reduction of IOP in different series. Table showing IOP in the preoperative period and one month after surgery. An IOP reduction varying from 0.5 to 3 mmHg is observed

Numerous studies have shown that cataract surgery by phacoemulsification with posterior chamber IOL induces a mid-and long-term lowering of IOP.[5‐10] Although elevations in IOP may occur in the immediate postoperative period due to retained viscoelastic material, the IOP is known to normalize within two to four hours.[11‐15] IOP decrease after CCP in normal patients has been demonstrated by Jahn,[7] Shingleton,[8] Tong and Miller.[10] They showed a reduction of IOP varying from 0.5 to 3 mmHg.[7,16‐19] In our study, the mean reduction was 2.25 mmHg (15.77%) [Figure 4]. Central corneal thickness was not assessed. Since IOP can vary considerably based on corneal thickness, this fact may be considered as a limiting factor of this study

IOP reduction has been shown to be more prominent after CCP than after phacoemulsification with sclerocorneal tunnel.[9,14] However, Tong and Miller found no significant difference in IOP changes between CCP and sclerocorneal phacoemulsification.[10] We are unable to comment on the effect of sclerocorneal incision as this technique was not employed by us. The reduction in IOP has been reported to last at least for a period of one year.[20,21] We followed our patients for a period of six months.

The exact mechanism by which cataract surgery improves IOP is unclear. Many hypotheses have been presented in the literature,[2] namely 1) hyposecretion of aqueous humor secondary to ciliary body irritation (CCP produces free radicals that may act as inflammatory mediators causing break down of the blood-aqueous barrier), 2) increased outflow of aqueous humor (CCP increases endogenous prostaglandins secretion rate that may augment uveoscleral outflow and consequently lower IOP), ultrasound stimulates the production of interleukins 1α by trabecular meshwork, increasing outflow facility and it may also be that the irrigation during phacoemulsification flushes the trabeculum, thereby decreasing outflow resistance.[2] and finally 3) improvement of aqueous outflow facility by widening effect of lens extraction on angle of AC. Cekic *et al* reported that the size of the capsulorhexis had an effect on the IOP after phacoemulsification; they showed that a capsulorhexis of 4 mm had a greater IOP lowering effect than a capsulorhexis of 6 mm.[22] In our series, a capsulorhexis of 5 mm was performed in all patients, but the effect of the size of the capsulorhexis in IOP could not be clearly demonstrated.

Studies on patients with open-angle glaucoma have demonstrated a pressure lowering effect of CCP.[5,18,23] In eyes with narrow angles, CCP increases the ACD and can permanently normalize IOP. In eyes with primary angle closure, cataract surgery attenuates the anterior positioning of the ciliary processes leading to significant widening of the angle. Corneal phacoemulsification has been recommended as an appropriate surgical procedure in a compliant glaucoma patient on 1 or 2 medications preoperatively with otherwise stable visual fields and optic nerve morphology.[2,18]

In conclusion, cataract surgery by CCP induces reduction of IOP in normal patients. The pathogenic mechanisms are still unclear. CCP can be performed with the intent of achieving better IOP control.
